# Polymorphism of nanocrystalline TiO_2_ prepared in a stagnation flame: formation of the TiO_2_-II phase†
†Electronic supplementary information (ESI) available: (1) Particle size and morphology, (2) FFT analysis of HRTEM images, and (3) XPS C 1s fitting. See DOI: 10.1039/c8sc02969e


**DOI:** 10.1039/c8sc02969e

**Published:** 2018-11-14

**Authors:** Manoel Y. Manuputty, Jochen A. H. Dreyer, Yuan Sheng, Eric J. Bringley, Maria L. Botero, Jethro Akroyd, Markus Kraft

**Affiliations:** a Department of Chemical Engineering and Biotechnology , University of Cambridge , West Site, Philippa Fawcett Drive , Cambridge , CB3 0AS , UK . Email: mk306@cam.ac.uk; b Cambridge Centre for Advanced Research and Education in Singapore (CARES) , CREATE Tower, 1 Create Way , 138602 , Singapore; c School of Chemical and Biomedical Engineering , Nanyang Technological University , 62 Nanyang Drive , 637459 , Singapore; d Department of Mechanical Engineering , National University of Singapore , 9 Engineering Drive 1 , 117575 , Singapore

## Abstract

A metastable “high-pressure” phase known as α-PbO_2_-type TiO_2_ or TiO_2_-II is prepared *via* a single-step synthesis using a laminar premixed stagnation flame. Three other TiO_2_ polymorphs, namely anatase, rutile and TiO_2_-B phases, can also be obtained by tuning the oxygen/fuel ratio. TiO_2_-II is observed as a mixture with rutile under oxygen-lean flame conditions. To the best of our knowledge, this is the first time that this phase has been identified in flame-synthesised TiO_2_. The formation of TiO_2_-II in an atmospheric pressure flame cannot be explained thermodynamically and is hypothesised to be kinetically driven through the oxidation and solid-state transformation of a sub-oxide TiO_2–*x*_ intermediate. In this scenario, rutile is nucleated from the metastable TiO_2_-II phase instead of directly from a molten/amorphous state. Mixtures containing three-phase heterojunctions of anatase, rutile, and TiO_2_-II nanoparticles as prepared here in slightly oxygen-lean flames might be important in photocatalysis due to enhanced electron–hole separation.

## Abstract 2

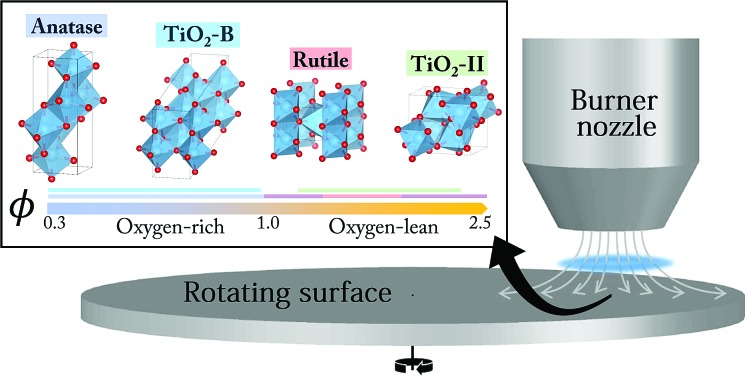
Flame-made TiO_2_ nanoparticles with tunable polymorphs, including the metastable TiO_2_-II phase, were prepared and a phase formation mechanism was proposed.

## 1 Introduction

Flame synthesis is widely used to manufacture functional metal oxide nanoparticles for applications including thermochemical analysis, chemical sensing, photocatalysis, and electrocatalysis.^1^,^2^ The advantages over other synthesis techniques are that the nanoparticles can be prepared through a one-step process^3^,^4^ and that the high temperature gradients inside the flame facilitate the formation of particles with unique properties.^5^ One such material that is frequently encountered in the field of flame synthesis is titanium dioxide.

A key attraction of flame-made TiO_2_ nanoparticles (TiO_2_-NPs) is the ability to readily tune properties such as particle size, aggregate morphology, and phase composition by controlling the synthesis conditions. These structural properties in turn control the catalytic activity of the formed TiO_2_-NPs.^6^,^7^ For example, the performance of TiO_2_ photocatalysts can be greatly enhanced by making use of anatase/rutile heterojunctions when compared to either pristine phase.^6^ Both anatase and rutile have been shown to form in a flame synthesis.^8^–^10^ Under certain conditions brookite has been observed although only in trace amounts.^11^ Recently, metastable phase TiO_2_-B^12^,^13^ has also been identified to form during flame synthesis.^14^ Various studies demonstrated that the phase composition of flame-made TiO_2_-NPs can be controlled by changing the oxygen/fuel equivalence ratio,^10^,^14^ TiO_2_ precursor loading,^8^,^10^ presence of an external electric field^15^ or laser irradiation,^16^ and dopant concentration.^17^

Three important factors affecting the stability of various TiO_2_ polymorphs have been identified, namely particle size, oxidising environment, and temperature. Zhang and Banfield^18^ demonstrated that for equally sized nanoparticles at 900–1000 K, anatase was the most stable phase for particles smaller than 11 nm, brookite for particles between sizes of 11 and 35 nm, and rutile for particles larger than 35 nm. For particles with similar size, the anatase–rutile composition was shown to be highly sensitive to the oxidant/reductant equivalence ratio, with rutile preferred in oxygen-lean and anatase in oxygen-rich flame environments.^8^,^10^ It was suggested that the formation of oxygen vacancies plays an important role in the rutile stabilisation. Recently Liu *et al.*^9^ expanded the thermodynamic analysis of Zhang and Banfield^18^ to include surface oxygen adsorption/desorption, which showed good agreement with their experimental observations. This treatment, however, was only applicable to the anatase and rutile system at thermodynamic equilibrium. Other studies have suggested that kinetically driven processes should be considered in the TiO_2_ phase transformation. For example, Mao *et al.*^19^ demonstrated a sintering-induced anatase-to-brookite transformation in 2–3 nm particles using molecular dynamics simulations.

In addition to the structures mentioned above, crystalline TiO_2_ can exist in other, less-studied, polymorphic forms such as TiO_2_-II (columbite) and TiO_2_-H (hollandite). TiO_2_-II, an orthorhombic high-pressure phase of TiO_2_ isostructural with α-PbO_2_, is of particular interest in this work. Although pure TiO_2_-II is only thermodynamically stable at high pressure conditions, experiments and first-principles studies have shown that it can be retained at ambient pressure as a metastable phase.^20^ TiO_2_-II has been found in nature as a mixture with rutile in ultra-high pressure metamorphic minerals.^21^,^22^ Zhao *et al.*^23^ suggested that small domains of TiO_2_-II could be stabilised at a three-phase anatase/TiO_2_-II/rutile junction. They predicted that such a three-phase junction will lead to a synergistic effect in mixed-phase TiO_2_ catalysts to enhance the electron–hole separation in photocatalysis. However, the role of TiO_2_-II as a photocatalyst is still inconclusive as the properties are strongly dependent on the synthesis routes used.^24^–^26^

Herein we demonstrate, for the first time, the formation of the TiO_2_-II phase at atmospheric pressure *via* stagnation flame synthesis in addition to the commonly observed phases anatase and rutile, as well as the previously reported metastable phase TiO_2_-B. The relative composition of these phases is strongly dependent on the oxygen/fuel ratio in the synthesis. The formation of metastable phases and their dependence on the oxidising environment give new insights into the phase formation and transformation mechanisms of TiO_2_-NPs in flames. The role of phase composition in the TiO_2_ photocatalytic activity is discussed further by Wu *et al.*^27^

## 2 Methods

### 2.1 Sample synthesis

The TiO_2_ nanoparticles (NPs) in this study were prepared with a premixed flame stabilised on a stagnation surface. A similar setup has been described in more detail elsewhere.^8^,^28^ Briefly, a mixture of ethylene, oxygen, and argon was ejected from a central aerodynamic nozzle with a total volumetric flow rate of 28 slpm. The nozzle had an exit diameter of 1.4 cm, resulting in an exit velocity of 436 cm s^–1^ at 150 °C. The nozzle shape induced a flat plug flow of premixed gas that impinged on a stagnation surface. Titanium tetraisopropoxide (TTIP, ≥97%, Sigma-Aldrich) was injected into the unburned gas mixture with a syringe pump at 8 ml h^–1^. The gas line, precursor line, and burner surface were heated to 150 °C to prevent TTIP condensation. During the experiment, the undoped flame was first stabilised for 15 minutes before TTIP was injected for 4 minutes. A shroud flow of 20 slpm N_2_ gas was used to stabilise the jet flow.

**Fig. 1:**
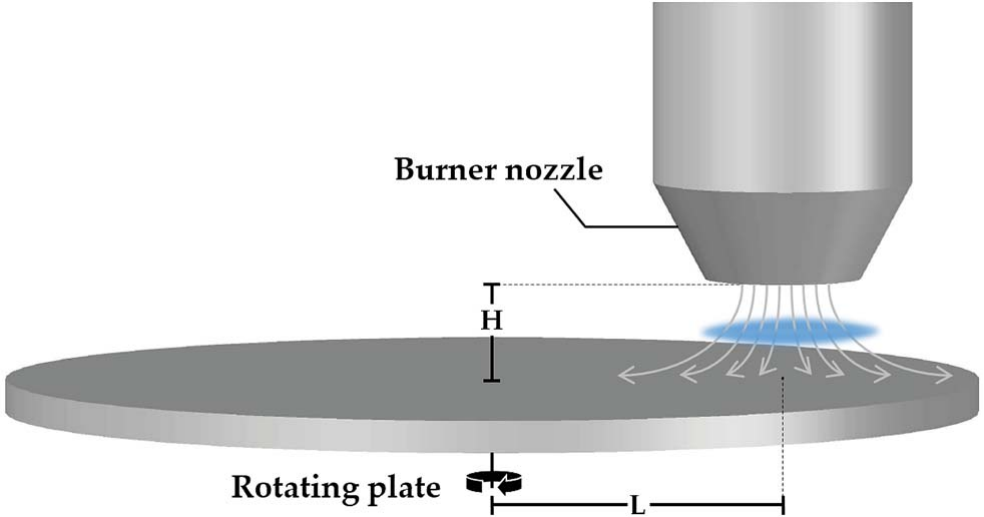
Schematic of the stagnation flame stabilised on a rotating plate (not drawn to scale). A burner nozzle is fixed at *H* = 1 cm above the rotating plate and at *L* = 10 cm from the rotational axis.

Two types of stagnation surfaces were located 1 cm under the nozzle to stabilise the flame by flow stretch and to accommodate a substrate for collecting the TiO_2_ sample. The first one was a rotating (300 rpm), circular stainless steel plate with its rotational axis located 10 cm from the burner centerline (Fig. 1). Slots in the stagnation surface enabled the positioning of glass substrates while the plate rotation convectively cooled the substrate and the deposited particles. In the second configuration, a water-cooled non-rotating plate was used as the stagnation surface. In both cases, a flat flame was stabilised 3–3.5 mm above the stagnation plate depending on the flame equivalence ratio. The equivalence ratio, *φ*, defined as the ratio of O_2_ required for the complete oxidation of introduced C_2_H_4_ divided by the actual amount of available O_2_, was varied as summarised in Table 1. After 4 min of TiO_2_ deposition, the sample was carefully scraped off from the glass substrate and used as prepared for further analysis.

**Table 1:** Flame mixture composition and the calculated flame adiabatic temperatures

*φ*	Mixture mole fractions			*T*_ad_ (K)
	C_2_H_2_	O_2_	Ar	
0.35	0.035	0.300	0.665	2073
0.50	0.035	0.210	0.755	2141
0.70	0.040	0.172	0.788	2327
0.80	0.040	0.150	0.810	2336
0.90	0.045	0.150	0.805	2437
1.00	0.050	0.150	0.800	2500
1.10	0.054	0.147	0.799	2518
1.30	0.061	0.141	0.798	2454
1.50	0.075	0.150	0.775	2422
1.67	0.103	0.185	0.712	2542
2.00	0.130	0.195	0.675	2402
2.30	0.172	0.224	0.604	2340
2.50	0.209	0.251	0.540	2309

### 2.2 Materials characterisation

Powder X-ray diffraction (XRD) patterns were recorded with a D8 Advance diffractometer (Bruker) with Cu K_α_ radiation (40 kV, 30 mA). The 2*θ* scan range was 20–90° with a step size of 0.02° and 3 s per step. Zero-background silicon sample holders were used with powder samples pressed to create a dense film.

X-ray photoelectron spectra (XPS) were recorded using a Kratos AXIS Ultra photoelectron spectrometer (Kratos Analytical Ltd.) fitted with a monochromatic Al K_α_ source (1486.71 eV, 5 mA, 15 kV). The photoelectrons were collected at an electron take-off angle of 90°. The binding energy shift was corrected by setting the C–C binding energy to 284.8 eV.

Transmission electron microscopy (TEM) images and selected area electron diffraction (SAED) patterns were acquired with a JEM-2100F FETEM (JEOL Ltd.) with 200 kV accelerating voltage. The TEM samples were prepared by applying a few drops of TiO_2_ suspension in ethanol on TEM grids followed by air-drying.

### 2.3 Simulation

The XRD patterns were simulated with BRASS^29^ using a simple isotropic size broadening model (Lorentzian) and experimental instrumental broadening parameters, assuming a zero background and 9 nm crystallite size. Instrumental broadening parameters were obtained experimentally with standard reference material 640e from NIST.

The undoped flames were simulated using *k*inetics®^30^ as one-dimensional stagnation flows coupled with detailed hydrocarbon chemistry described by the USC-Mech II model.^31^ The flame standing location was estimated to be 3.5 mm from the stagnation surface. The stagnation surface temperature was taken to be 420 K. A more detailed description of the simulation has been given elsewhere.^32^ Constant-volume equilibrium simulations at 1 atm and 150 °C were performed using *k*inetics® to estimate the flame adiabatic temperature (summarised in Table 1). It is noted that the flame adiabatic temperature is usually slightly higher than the actual flame temperature as there is convective heat loss to the colder stagnation plate.

## 3 Results and discussion

### 3.1 Particle morphology

**Fig. 2:**
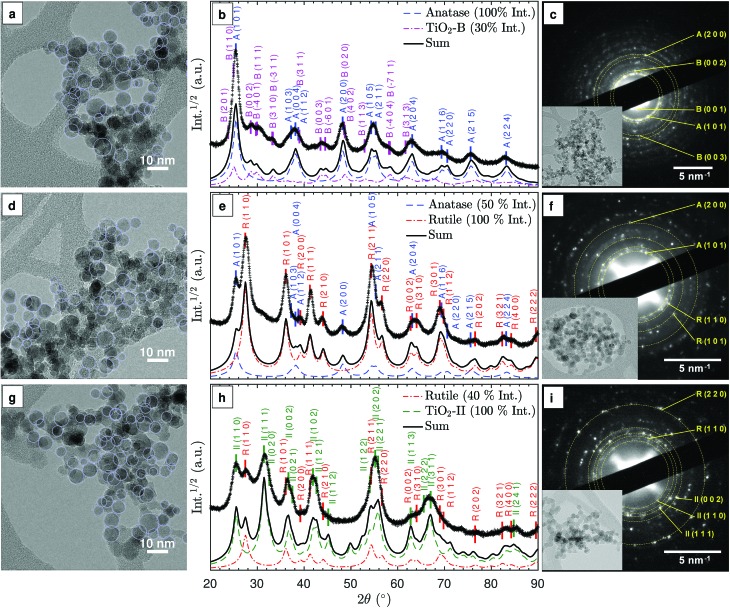
Characterisation of particles prepared under fuel-lean (top row, a–c), stoichiometric (middle row, d–f), and fuel-rich (bottom row, g–i) flame conditions. Left column: annotated TEM images showing spherical primary particles. Middle column: experimental (+) and simulated (lines, see text for details) XRD patterns for qualitative comparison. The locations of the main diffraction peaks for different TiO_2_ polymorphs (ICSD catalogue) are annotated and marked with vertical ticks. Right column: selected area electron diffraction (SAED) patterns with labels for the main diffraction spots and the corresponding TEM images as the inset.

The as-synthesised particles form agglomerates consisting of nearly spherical primary particles as highlighted in the TEM images in Fig. 2(a), (d), and (g). Similar particle shapes and sizes were observed in previous studies.^8^,^28^ Given the small particle residence time in the flame, the agglomerates are likely formed during particle deposition and TEM sample preparation. The average primary particle diameter, *d[combining macron]*_V_, is approximately 9 nm (see ESI Fig. S1†). No significant difference in *d[combining macron]*_V_ is observed with varying equivalence ratio despite approximately 500 K maximum variation in the adiabatic flame temperature (Table 1). The insensitivity of the particle size to the maximum flame temperature could be explained by the reduced particle residence time in hotter flames due to an increased convective velocity. Simulated temperature profiles and particle residence times demonstrating the compensating effect are included in the ESI (Fig. S2†).

### 3.2 Qualitative phase identification

The TiO_2_ phases that can be produced with the investigated flames were identified for three representative synthesis conditions: fuel-lean (*φ* = 0.50), stoichiometric (*φ* = 1.00), and fuel-rich (*φ* = 1.67). Fig. 2(b), (e), and (h) show the XRD patterns for these conditions. For the purpose of qualitative comparison, simulated XRD patterns were produced with a simple isotropic size broadening model (Lorentzian) and experimental instrumental broadening parameters, assuming no background and 9 nm crystallites based on the observed primary particle size (Fig. S1†). A complete Rietveld refinement of the XRD patterns was not possible due to the complex mixtures of nano-sized crystals. It is suspected that the rapid sample quenching introduced additional peak broadening through micro-strain (see below) and possibly some degree of anisotropy in the strain and size induced broadening. In the following discussion, prefixes A, R, B and II denote anatase, rutile, TiO_2_-B and TiO_2_-II crystal phases, respectively.

In the lean flame (*φ* = 0.50, Fig. 2(b) and (c)), the major diffraction peaks can be ascribed to anatase (ICSD no. 92363), *e.g.*, A(1 0 1) at 25.3° and A(2 0 0) at 48.1°. The presence of TiO_2_-B (ICSD no. 41056) can be identified from the smaller peaks at 2*θ* = 27–34°, *i.e.* B(0 0 2), B(1 1 1), B(3 1 0). The simulated XRD pattern with a 100 : 30 intensity ratio of anatase to TiO_2_-B correctly predicts all the main peaks observed in the experimental pattern. The simulated 2*θ*-dependent peak broadening due to the crystal size is insufficient to reproduce the broad peaks observed at high angles, *e.g.*, A(2 1 5) and A(2 2 4), suggesting additional peak broadening due to micro-strain.

In the stoichiometric flame (*φ* = 1.00, Fig. 2(e) and (f)), rutile (ICSD no. 16636) was formed in addition to anatase, as is evident from the R(1 1 0), R(1 0 1), R(1 1 1), and R(2 1 1) peaks. The presence of a broad shoulder at 2*θ* = 31° potentially originates from a third phase, such as a small amount of TiO_2_-B or TiO_2_-II. The qualitative agreement observed between the experimental and simulated XRD patterns confirms that rutile and anatase are the main polymorphs formed in the stoichiometric flame (at approximately 2 : 1 intensity ratio).

In the rich flame (*φ* = 1.67, Fig. 2(h) and (i)), a significant peak at 2*θ* = 31.5° is observed, which is consistent with II(1 1 1) planes of the TiO_2_-II polymorph (ICSD no. 158778). It is noted that the peak at 25.5° can either be ascribed to A(1 0 1) or II(1 1 0) but the lack of the A(2 0 0) peak at 48° suggests that the latter is the case. In addition, rutile can be identified from the R(1 1 0) peak at 27.5°. Comparison between the simulated and measured XRD patterns shows slightly broader experimental peaks at low 2*θ*, indicating that the measured crystals are smaller than the 9 nm assumed for the simulated XRD. The difference in peak broadening increases with 2*θ*, suggesting the presence of additional micro-strain. Furthermore, it can be observed that some peaks such as II(1 1 2) at 44.5° and II(1 1 3) at 62.5° are significantly smaller and/or broader than expected, most likely due to anisotropy in the crystals. It is interesting to note that similar XRD patterns with strong anisotropy in size and strain-induced broadening were observed in rutile and TiO_2_-II formed through high-energy milling experiments.^24^,^25^

For all three flames, the selected area electron diffraction (SAED) patterns of the agglomerated particles (Fig. 2(c), (f), and (i)) are consistent with the powder XRD patterns. The presence of TiO_2_ phase mixtures on an aggregate scale suggests intimately mixed crystals at the particle level. For samples prepared in lean flames, the presence of diffraction spots with approximately 0.63 nm lattice spacing corresponding to the B(0 0 1) planes confirms the presence of the TiO_2_-B phase (Fig. 2(c)).

**Fig. 3:**
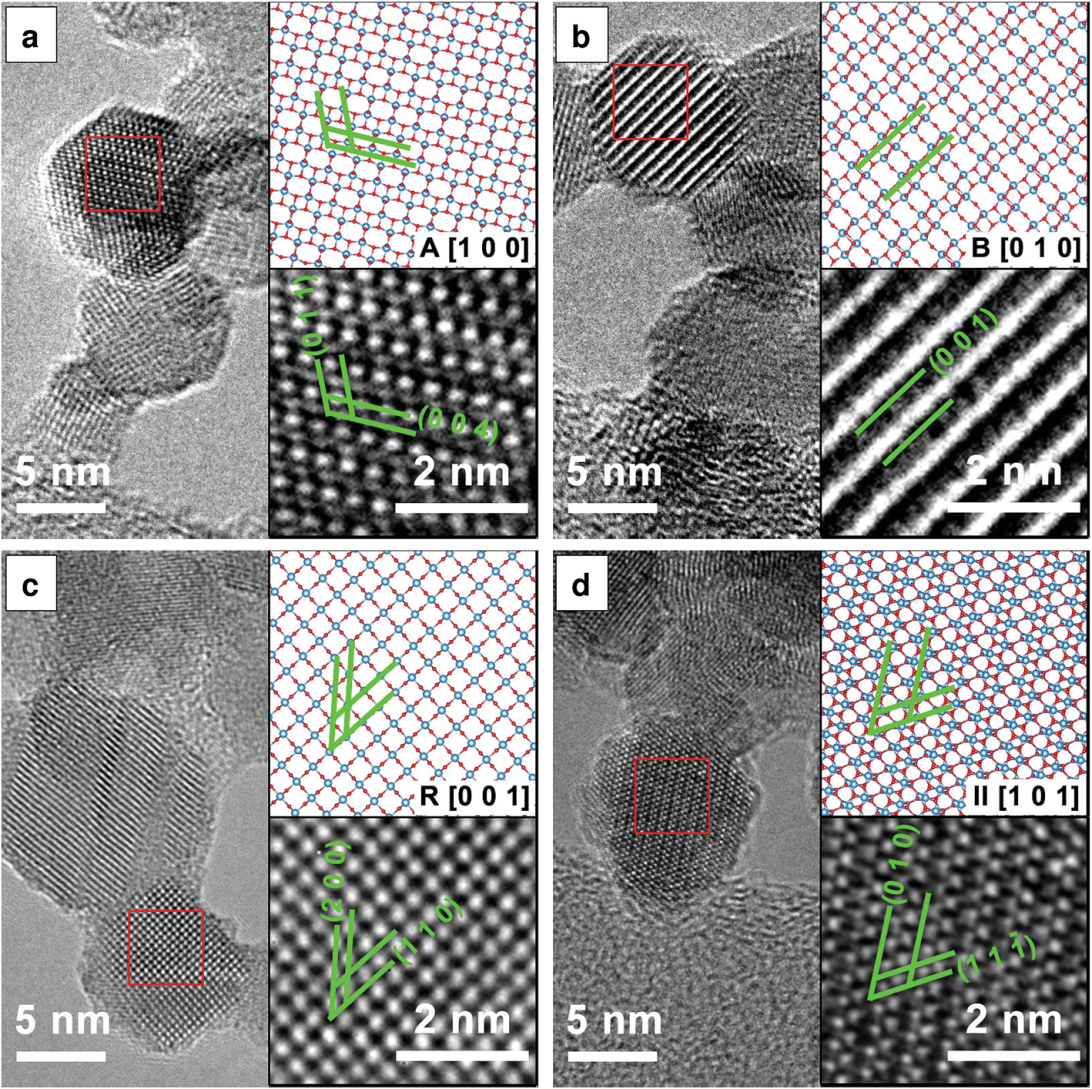
HRTEM images of the four TiO_2_ polymorphs identified at different flame equivalence ratios *φ*: (a) anatase, *φ* = 0.50, (b) TiO_2_-B, *φ* = 0.50, (c) rutile, *φ* = 1.00, (d) TiO_2_-II, *φ* = 1.67. The bottom inset is an enlarged view of the area in the red square. The top inset is a ball-and-stick representation of the particular polymorph with the same orientation as in the HRTEM image (Ti: blue, O: red). The relevant crystal planes are annotated in green.

Fig. 3 presents the HRTEM images and the corresponding structural models which further confirm the presence of the anatase, rutile, TiO_2_-B and TiO_2_-II phases as discussed previously. The lattice spacings measured from the HRTEM images agree with those from the ICSD data to within 5% accuracy (a reasonable uncertainty expected from TEM^33^). In particular, TiO_2_-B can be readily identified by the large spacing of B(0 0 1) planes as shown in Fig. 3(b) (*d* = 6.32 Å, ref. *d*_B(001)_ = 6.24 Å). In Fig. 3(d), lattice planes corresponding to II(1 1 1[combining macron]) (*d* = 2.86 Å, ref. *d*_II(1 1 1[combining macron])_ = 2.85 Å) and II(0 1 0) (*d* = 5.63 Å, ref. *d*_II(010)_ = 5.50 Å) are marked. The measured interplanar angle is 60.5° (ref. *α*_II(1 1 1[combining macron])/(0 1 0)_ = 58.8°).

While Fig. 3 indicates the presence of single crystal primary particles, other TEM images show stacking faults and possibly even multiple crystals within single primary particles (see Fig. S3†). These different crystal domains are especially evident in particles prepared in the fuel-rich flame (*φ* = 1.67), explaining the anisotropy observed in the powder XRD patterns (Fig. 2(h)). Unfortunately, it was not possible to confidently determine the actual crystal phases, orientations and boundaries or their epitaxial relationships, if any, in a single primary particle.

### 3.3 Effects of flame equivalence ratio

**Fig. 4:**
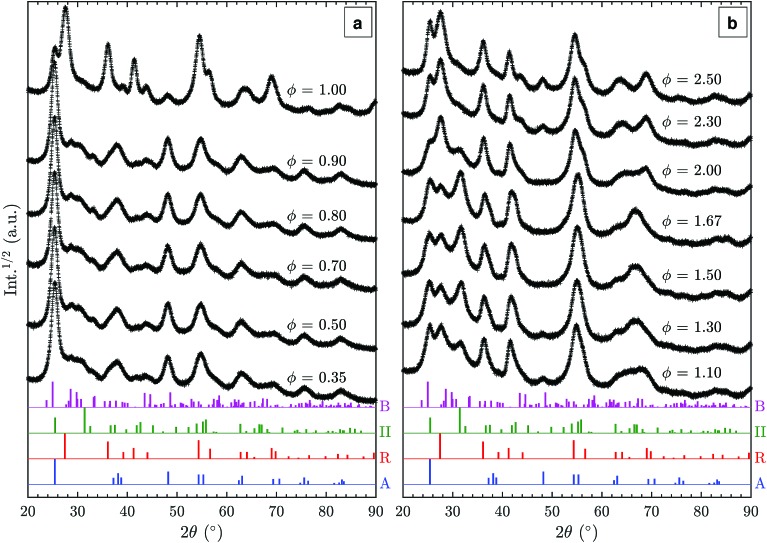
Powder XRD patterns of particles prepared at varying flame mixture equivalence ratios, *φ*: (a) fuel-lean and (b) fuel-rich flames. Reference XRD patterns of the different polymorphs from the ICSD catalogue are given at the bottom (A: anatase, B: TiO_2_-B, R: rutile, II: TiO_2_-II).

Additional XRD patterns of samples prepared in fuel-lean (*φ* < 1.0), stoichiometric (*φ* = 1), and fuel-rich (*φ* > 1.0) flames were measured to elucidate the effect of fuel/oxygen equivalence ratio *φ* on the formed TiO_2_ polymorphs (Fig. 4). In the range of *φ* = 0.35–0.90, no substantial change is observed despite the significant variations in adiabatic flame temperatures of up to 400 K (Table 1). The particles are predominantly anatase with some amount of TiO_2_-B as discussed in Section 3.2. Using a rotating stagnation plate, very similar to the one used in this study, Memarzadeh *et al.*^8^ and Liu *et al.*^9^ observed only anatase in lean flames (*φ* = 0.45–0.9) with a small amount of rutile. McCormick *et al.*^11^ reported the formation of anatase with a minor amount of a metastable phase identified as brookite prepared in a *φ* = 0.36 flame followed by annealing at 773 K. This discrepancy likely arises from varying stagnation surface temperature in these studies, leading to different quenching rates compared to this work. For example, Riad *et al.*^14^ recently reported, for the first time, the formation of the metastable TiO_2_-B phase (27% as a mixture with anatase and amorphous phases) in samples prepared through flame spray pyrolysis (FSP) synthesis. In their study, TiO_2_-B was preferentially formed under oxygen-rich conditions, consistent with the observations made in this work. As increasing oxygen content leads to shorter residence time in the FSP, it is suggested that the short residence time is responsible for the formation of TiO_2_-B.

Near the stoichiometric point (*φ* = 0.90–1.00), anatase and rutile are the two main phases observed. The rutile content increases with increasing equivalence ratio as is evident from the R(1 1 0) peak at 27.5°. An anatase/rutile transition near the stoichiometric point was also observed by Liu *et al.*^9^ The strong dependence of the anatase/rutile ratio on the equivalence ratio is further in agreement with the study by Kho *et al.*^10^

As the equivalence ratio is increased further (*φ* = 1.00–1.30), the XRD results indicate that the anatase content decreases (*i.e.* A(2 0 0) peak at 48.1°) while TiO_2_-II is formed. It is noted that the XRD results obtained by Liu *et al.*^9^ at *φ* = 1.15 and 1.33 also showed a peak at 2*θ* = 31.5° but it was attributed to the presence of impurity (Ti_3_O_5_). As discussed previously, it is suggested that this peak originates from the II(1 1 1) reflection instead. It should also be noted that Liu *et al.*^9^ assigned the reflection at 25.3° to A(1 0 1) but it might also be caused by II(1 1 0), which would explain the absence of the A(2 0 0) reflection in their pattern. The higher intensity of the 2*θ* = 31.5° peak in the present study is likely caused by differences in the synthesis conditions such as the deposition time, actual gas flow rates, stagnation surface temperature, or burner nozzle diameter.

In the *φ* range of 1.50–1.67, the two main phases identified are rutile and TiO_2_-II with significant amounts of the latter. The as-synthesised powders appeared slightly blue suggesting the presence of lattice oxygen deficiencies.^34^ To the best of our knowledge, our work is the first to report a substantial amount of TiO_2_-II prepared through flame synthesis. A detailed discussion on the possible formation routes is given below.

For the very-fuel-rich flame condition (*φ* = 2.00–2.50), soot is formed together with TiO_2_ causing the obtained powder to be coloured grey-black. Based on the XRD pattern (Fig. 4(b)), rutile and TiO_2_-II are still present but with a higher content of rutile. Additionally, anatase is formed as the equivalence ratio increases above 2.0 as evidenced by the A(2 0 0) peak at 48.1°.

**Fig. 5:**
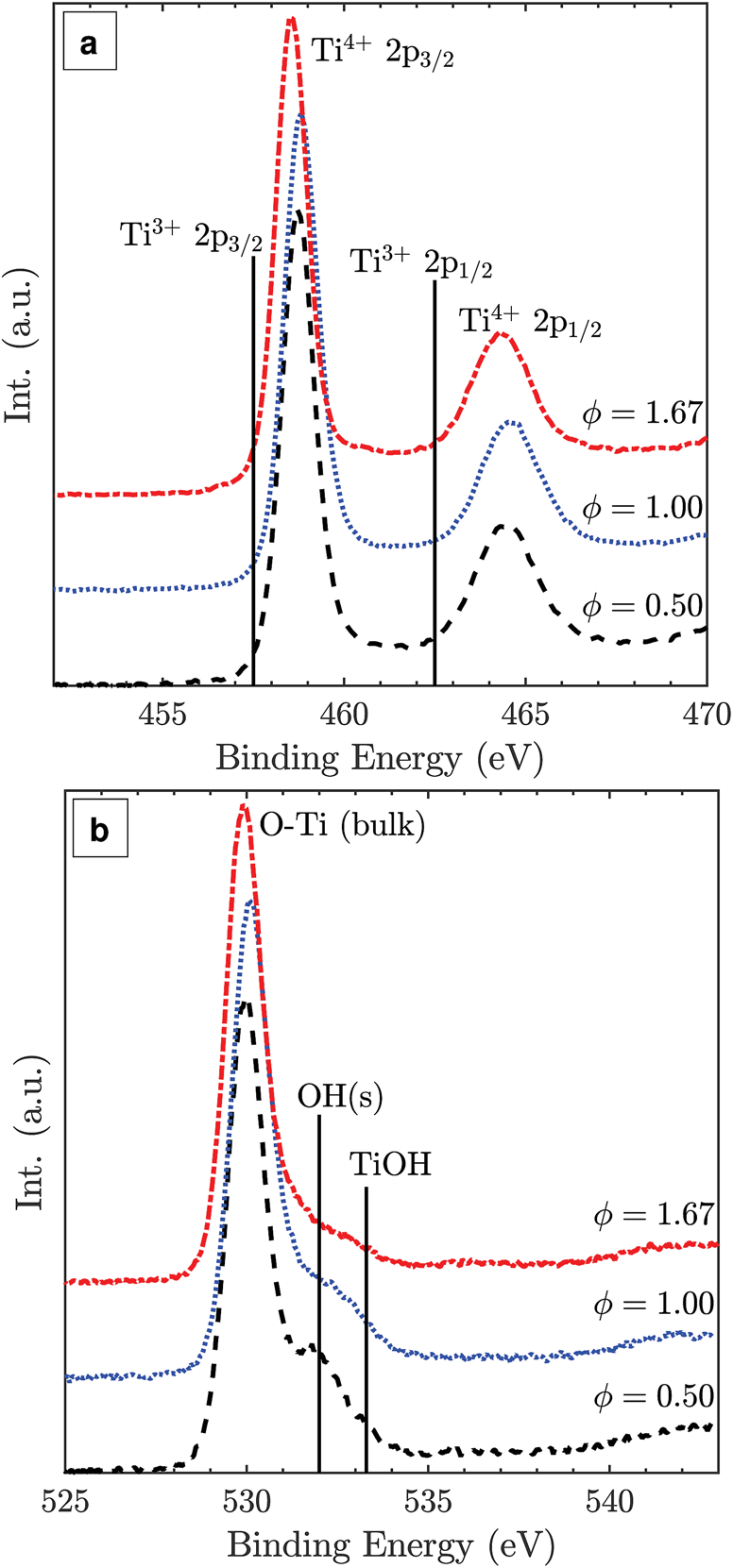
Ti 2p (a) and O 1s (b) XPS spectra of TiO_2_ particles synthesised at lean, stoichiometric, and rich flame conditions with normalised intensities.

The Ti 2p XPS spectra of samples from the lean, stoichiometric, and rich flames (Fig. 5(a)) show very similar binding energies and intensities for Ti^4+^ 2p_3/2_ and Ti^4+^ 2p_1/2_ peaks with a 2p_3/2_–2p_1/2_ splitting value of 5.8 eV, consistent with reported values for TiO_2_.^35^ No detectable Ti^3+^ presence is observed suggesting that the particle surface is completely oxidised regardless of the difference in the oxygen environment (positions of Ti^3+^ binding energies are marked for reference in Fig. 5(a)).^36^ The main O 1s peak at around 530.1 eV can be assigned to bulk oxygen in TiO_2_ (Fig. 5(b)). The smaller peaks at higher binding energies likely belong to surface oxygens, the acidic OH(s) and the basic TiOH, formed from H_2_O chemisorption on the surface.^37^

### 3.4 Formation of the TiO_2_-II polymorph

In the rich flames where TiO_2_-II is formed (*φ* = 1.10–2.00), the calculated adiabatic flame temperature is 2400–2500 K (Table 1). At this temperature, incipient particles in the flame are likely to melt or be liquid-like without any long range order^38^ (melting point of bulk TiO_2_ approx. 2100 K). The particles grow in size through surface growth and coalescence until they approach the stagnation surface where they are rapidly cooled and solidify (Fig. S2†). The presence of both TiO_2_-II and rutile in these samples most likely indicates that one of them formed first and that the other developed through a phase transformation. One possibility is the formation of solid rutile particles followed by a solid-state transformation to TiO_2_-II. Another option would be the direct formation of TiO_2_-II (or a pre-TiO_2_-II intermediate phase) and a subsequent solid-state transformation to rutile. Both scenarios are considered here and will be discussed below.

Numerous studies have documented solid-state transformations of rutile, anatase, or brookite to TiO_2_-II but this typically requires high-pressure conditions of up to 5–9 GPa.^39^,^40^ Such a high pressure can be achieved through static pressing, shock wave, or high-energy milling experiments. As the TiO_2_-II in the present study was formed in an atmospheric pressure flame, a solid-state transformation of rutile to TiO_2_-II is considered unlikely.

**Fig. 6:**
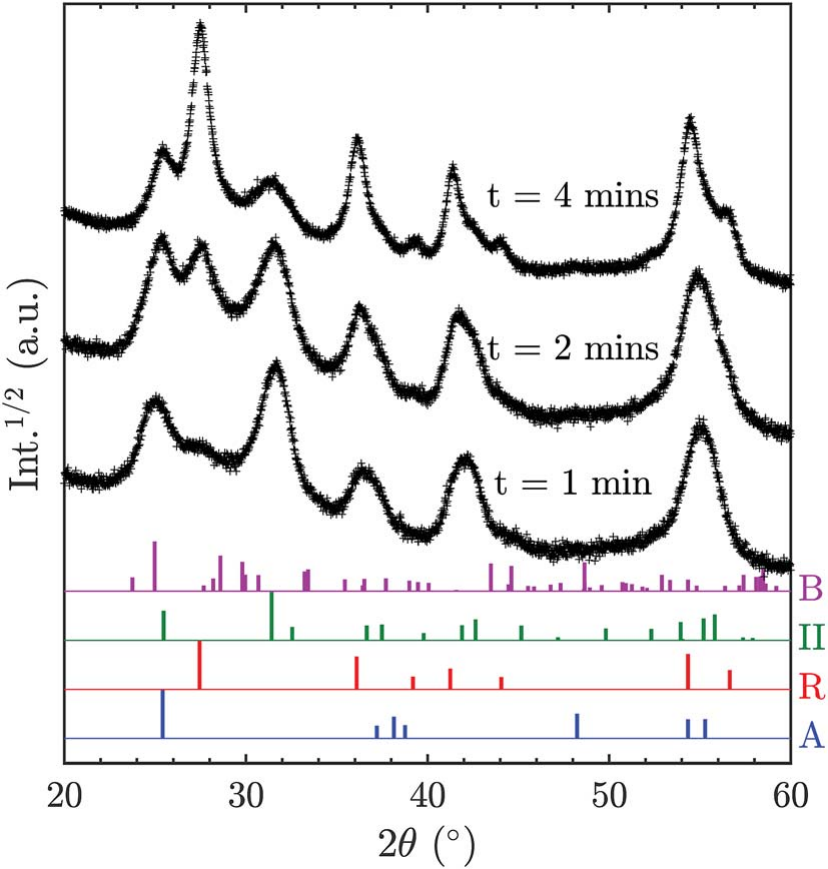
Powder XRD patterns of particles prepared at an equivalence ratio, *φ* of 1.67 with a non-rotating stagnation plate at varying deposition time, *t*. Reference XRD patterns of the different polymorphs from the ICSD catalogue are given at the bottom (A: anatase, B: TiO_2_-B, R: rutile, II: TiO_2_-II).

In order to elucidate the possibility of direct TiO_2_-II formation with subsequent phase transformation to rutile, additional XRD patterns (Fig. 6) were recorded for samples collected for different durations on a water-cooled plate (instead of a rotating stagnation plate). Note that the surface temperature of the stationary plate is likely to be higher than that of the rotating plate; thus the results are not directly comparable. Nevertheless, the change of phase composition with prolonged deposition time can give valuable information on the origin of the TiO_2_ polymorphs. It can be observed in Fig. 6 that the rutile content increases with increasing deposition time and thus prolonged exposure to elevated temperatures. This suggests that TiO_2_-II is formed first and that rutile originates from a solid-state phase transformation of the already deposited TiO_2_-II particles. Furthermore, the solid-state transformation is consistent with the observed multiple crystal domains within a single primary particle (Fig. S3†) and the accompanying crystal anisotropy.

A comparison to other ambient pressure TiO_2_-II synthesis routes might help explain the role of the oxygen/fuel equivalence ratio on the TiO_2_-II formation and the possible involvement of pre-TiO_2_-II intermediates. Aarik^41^ used atomic layer deposition (ALD) to grow TiO_2_-II solid films from a TiCl_4_ gas-phase precursor and water as the sole oxygen source. It was observed that TiO_2_-II grows with some preferred orientation in the pure crystalline phase or in a mixture with rutile at low water doses (*i.e.* oxygen-lean environment),^42^ in agreement with our findings for fuel-rich (*i.e.* oxygen-lean) flames. A preferred growth orientation for TiO_2_-II was also observed by Grey *et al.*^43^ who reacted TiO_2_ sub-oxide (with composition close to Ti_3_O_5_) with boiling sulphuric acid. They proposed that TiO_2_-II was formed through a solid state transformation of an α-Ti_3_O_5_ due to a small long-range misfit between the atomic arrangements of α(1 1 0) and II(1 0 1) layers. Therefore, the results from Aarik *et al.*^42^ and Grey *et al.*^43^ demonstrate the importance of a non-stoichiometric surface layer^42^ or solid state transformation from α-Ti_3_O_5_ sub-oxide in the formation of TiO_2_-II crystals. Such sub-oxide species can potentially be formed in the gas phase through clustering of species such as Ti and TiO which have recently been identified as important products in the TTIP decomposition.^44^ Similarly, sub-oxide structures such as Ti_3_O_5_ and Ti_5_O_7_ have been reported to form during plasma synthesis of TiO_2_ from TiC oxidation.^45^ Therefore, it is possible that during flame synthesis, sub-oxide species form directly from Ti and TiO clustering in the fuel-rich flames. In the high temperature flame environment, these sub-oxide clusters would continue to grow in a liquid-like state and at the same time be oxidised to form stoichiometric TiO_2_. With high cooling rates, it is possible that the sub-oxide clusters solidify prior to complete oxidation. In this case, diffusion of lattice oxygen can occur to further oxidise the TiO_2_ bulk, kinetically favouring the formation of TiO_2_-II over rutile through a mechanism similar to that described by Grey *et al.*,^43^*i.e.* solid-state transformation driven by the close structural match between (1 1 0) α-Ti_3_O_5_ and (1 0 1) TiO_2_-II planes.

If sub-oxide species indeed solidified and later oxidised to TiO_2_-II through the diffusion of lattice oxygen, some residue of Ti^3+^ could be expected. Such oxygen deficient titania was reported to be blue in colour,^34^ similar to the colour of the particles synthesised here with the fuel-rich flames. Notably, the presence of some Ti^3+^ or oxygen vacancies in the particle core does not contradict the absence of a Ti^3+^ peak in the surface sensitive XPS spectra (Fig. 5). Rather, it is assumed that the particles have a completely oxidised surface as confirmed by XPS but some oxygen vacancies or Ti^3+^ exist in the core resulting in the blue coloration of the powder.

Surface modifications could also possibly explain the TiO_2_-II formation as they can strongly affect the energetics of nanocrystals.^9^,^46^ For example, Barnard and Zapol^47^ show that the anatase-to-rutile phase transition size is significantly affected by surface passivation. The effect of surface hydration has been demonstrated for Y_2_O_3_ polymorphic stability where nano-sized particles with a metastable high-pressure polymorph have been prepared at ambient pressure.^48^,^49^ The XPS data in Fig. 5(b) suggest that there are some qualitative differences in the shape and intensity of the surface oxygen peaks. However, the interpretation of these peaks is beyond the scope of the present study and will be investigated in future work.

Lastly, the decrease in the relative ratio of TiO_2_-II to rutile and the onset of anatase formation in very rich flames (*φ* = 2.00–2.50, Fig. 4(b)) is likely connected to the soot formation at these conditions. The formation of soot is evident from the gray-black coloration of the collected powder and is expected to decrease the flame temperature due to radiative heat loss and might further affect the gas-phase chemistry.

## 4 Conclusions

Four different TiO_2_ polymorphs and their mixtures were prepared with a single synthesis step using a premixed laminar stagnation flame. Metastable phases TiO_2_-B and TiO_2_-II were identified from diffraction and microscopy analysis. The obtained crystal structure was controlled by varying the fuel/oxygen equivalence ratio, *φ*, where mixtures of anatase/TiO_2_-B, anatase/rutile, and rutile/TiO_2_-II were formed under fuel-lean, stoichiometric, and fuel-rich conditions, respectively.

Notably, this is the first time that TiO_2_-II is reported to form in atmospheric pressure flames even though similar XRD patterns were observed in previous flame studies. The mechanism leading to the formation of rutile/TiO_2_-II mixtures in fuel-rich (*i.e.* oxygen-lean) flames was elucidated based on the phase composition as a function of collection time and previous reports of TiO_2_-II formation. It is proposed that rutile is formed through a solid-state transformation of TiO_2_-II. The TiO_2_-II formation hereby likely involves some titania sub-oxide intermediate that is subsequently oxidised to stoichiometric TiO_2_-II. The formation of and transformation between the four different TiO_2_ polymorphs cannot be explained by the current understanding of TiO_2_ phase formation and transformation mechanism, and thus requires re-evaluation of the current working hypotheses.

## Conflicts of interest

The authors declare no conflicts of interest.

## Supplementary Material

Supplementary information
