# Supplementary material for: Polymorphism of nanocrystalline TiO2 prepared in a stagnation flame: formation of the TiO2-II phase
Source: Chem Sci. 2018 Nov 14;10(5):1342–50. doi: 10.1039/c8sc02969e (PMC6354738; doi:10.1039/c8sc02969e)
Supplement: Supplementary file 1 [file SC-010-C8SC02969E-s001.pdf]

Electronic Supplementary Material (ESI) for Chemical Science.
This journal is © The Royal Society of Chemistry 2018

Supplementary Material (SM) for:

Polymorphism of nanocrystalline TiO_2 prepared in a stagnation flame: Formation of TiO_2-II phase

Manoel Y. Manuputty[a,b], Jochen A. H. Dreyer[a,b], Yuan Sheng[b,c], Eric J. Bringley[a], Maria L. Botero[b,d], Jethro Akroyd[a,b], Markus Kraft[a,b,c,*]

[a] *Department of Chemical Engineering and Biotechnology, University of Cambridge, West Site, Philippa Fawcett Drive, Cambridge, CB3 0AS, United Kingdom*
[b] *Cambridge Centre for Advanced Research and Education in Singapore (CARES), CREATE Tower, 1 Create Way, 138602, Singapore*
[c] *School of Chemical and Biomedical Engineering, Nanyang Technological University, 62 Nanyang Drive, 637459, Singapore*
[d] *Department of Mechanical Engineering, National University of Singapore, 9 Engineering Drive 1, 117575, Singapore*

---

[*] Corresponding author
Email address: mk306@cam.ac.uk (Markus Kraft)

1. Particle size and morphology

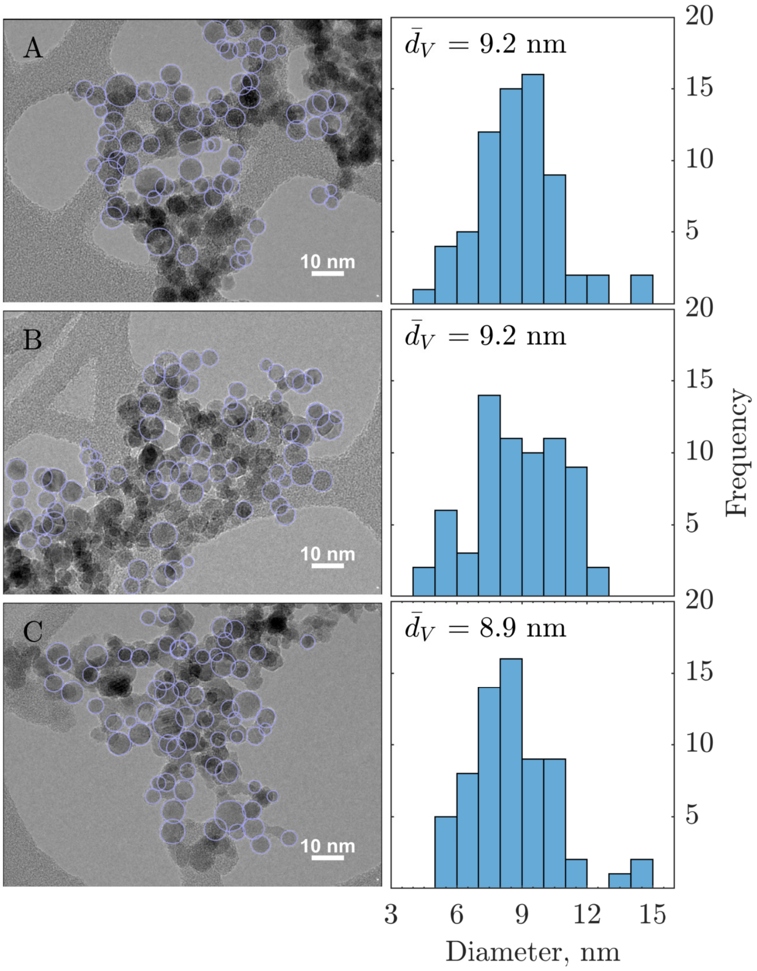

Figure S1: Annotated TEM images (left panel) of particles prepared in lean (A, $\phi = 0.50$), stoichiometric (B, $\phi = 1.00$), and rich (C, $\phi = 1.67$) flames. Particles size histograms (right panel) and the volume averaged particle size, $\bar{d}_V$, are from manual measurements of approximately 70 spherical primary particles on each TEM image; standard deviation, SD = 2.0 nm (A), 2.0 nm (B), 2.2 nm (C).

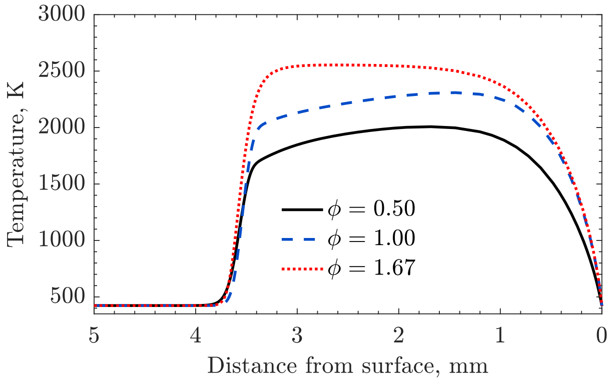

(a) Temperature profiles

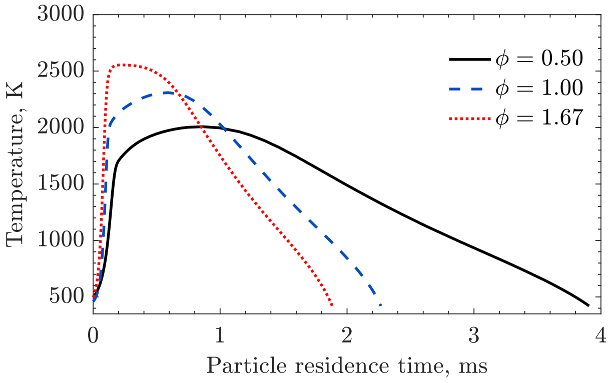

(b) Temperature-time profiles

Figure S2: Simulated particle temperature and residence time profiles in undoped lean ($\phi = 0.50$), stoichiometric ($\phi = 1.00$), and rich ($\phi = 1.67$) flames. Particles are assumed to be formed at $T \approx 500$ K ($t_{\mathrm{res}} = 0$). The residence time is calculated from convective and thermophoretic velocity experienced by particles. The profiles demonstrate that the particle residence time decreases as the flame temperature increases. This is expected as higher temperature leads to more gas expansion and subsequently higher convective velocity. This compensating effect explains the close particle sizes observed in Fig. S1

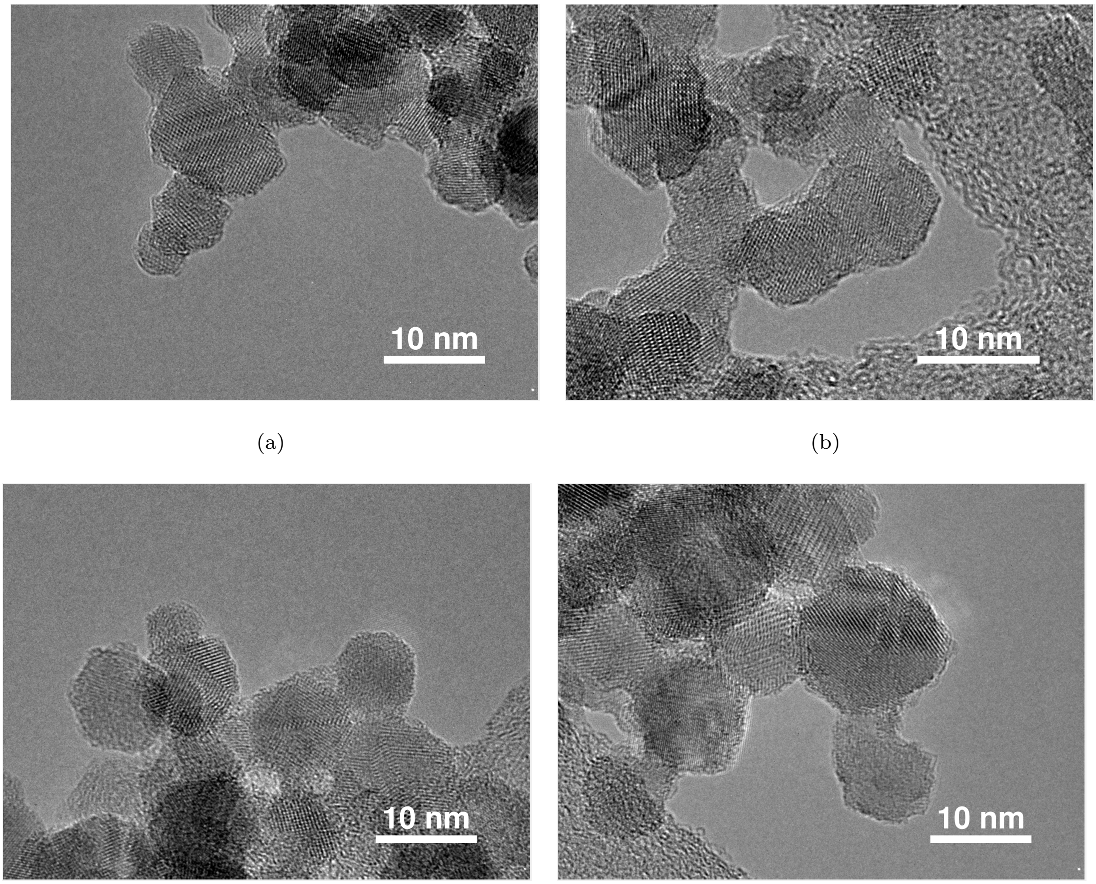

(a) (b)

(c) (d)

Figure S3: HRTEM images showing primary particles with possibly multiple crystal domains (not indexed) prepared in flames with equivalence ratio, ϕ, of 1.67 (a, b) and 1.00 (c, d).

2. FFT analysis of HRTEM images

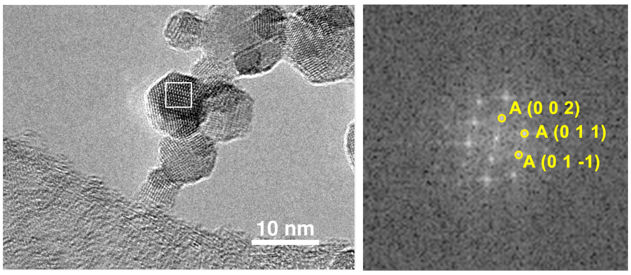

(a) Anatase ($\phi = 0.50$)

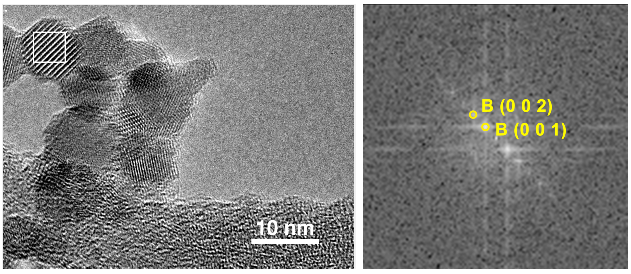

(b) TiO_2-B ($\phi = 0.50$)

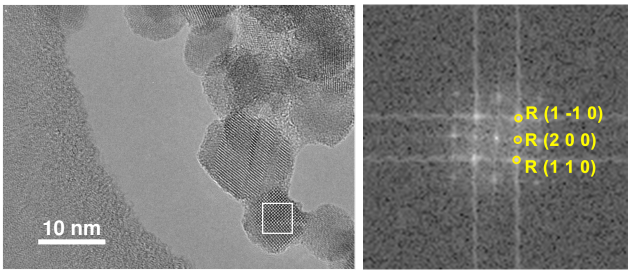

(c) Rutile ($\phi = 1.00$)

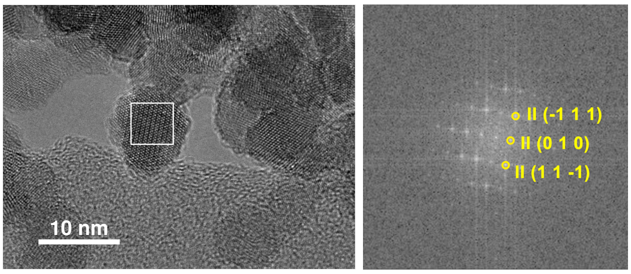

(d) TiO_2-II ($\phi = 1.67$)

Figure S4: HRTEM images (left) and the corresponding annotated FFT spectra (right). The summary of FFT analysis is given in Table S2.

Table S1: Lattice spacing, d, and the interplanar angles (relative to the first plane), α, obtained from FFT analysis of HRTEM images shown in Fig. S4. The reference data are taken from ICSD catalogue (see text for details on catalogue number for each phase).

Image	Measured (FFT)		Reference (ICSD)		
	d, Å	Relative α, °	Lattice plane	d, Å	Relative α, °
S4(a)	3.67	0	A $(0\ 1\ \bar{1})$	3.50	0
	3.56	44.5	A (0 1 1)	3.50	43.6
	4.82	113.6	A (0 0 2)	4.72	111.8
S4(b)	6.32	0	B (0 0 1)	6.24	0
	3.15	0.4	B (0 0 2)	3.12	0
S4(c)	3.38	0	R (1 1 0)	3.25	0
	2.35	43.5	R (2 0 0)	2.30	45
	3.34	92.4	R $(1\ \bar{1}\ 0)$	3.25	90
S4(d)	2.86	0	II $(1\ 1\ \bar{1})$	2.85	0
	5.63	60.5	II (0 1 0)	5.50	58.8
	2.87	117.9	II $(\bar{1}\ 1\ 1)$	2.85	117.7

3. XPS C 1s fitting

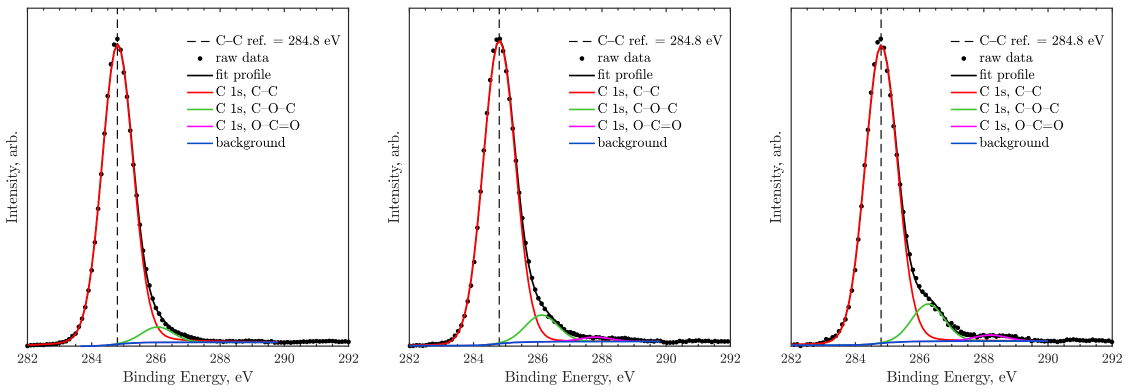

(a) $\phi = 0.50$ (b) $\phi = 1.00$ (c) $\phi = 1.67$

Figure S5: C 1s XPS spectra and fitted peaks of samples prepared in lean, stoichiometric, and rich flames.

Table S2: C-1s binding energies from XPS spectra deconvolution for internal calibration (C–C binding energy set at 284.8 eV). The spectra are fitted with constraints of 1) equal FWHM, and 2) 100% Gaussian shape for C–O–C and O–C=O peaks.

ϕ	Binding energy, eV			χ^2
	C–C	C–O–C	O–C=O	
0.50	284.8	286.1	-	3.65
1.00	284.8	286.1	287.8	5.29
1.67	284.8	286.3	288.2	4.81
